# 2-[(2-Hy­droxy­benz­yl)amino]­pyrazinium perchlorate–2-[(pyrazin-2-yl­amino)­meth­yl]phenol (1/1)

**DOI:** 10.1107/S1600536812031558

**Published:** 2012-07-18

**Authors:** Shan Gao, Seik Weng Ng

**Affiliations:** a Key Laboratory of Functional Inorganic Material Chemistry, Ministry of Education, Heilongjiang University, Harbin 150080, People’s Republic of China; b Department of Chemistry, University of Malaya, 50603 Kuala Lumpur, Malaysia; c Chemistry Department, Faculty of Science, King Abdulaziz University, PO Box 80203 Jeddah, Saudi Arabia

## Abstract

In the crystal structure of the title co-crystal, C_11_H_12_N_3_O^+^·ClO_4_^−^·C_11_H_11_N_3_O, the perchlorate ion is disordered about a twofold rotation axis with the Cl atom located on the twofold rotation axis; the 2-[(2-hy­droxy­benz­yl)amino]­pyrazinium cation and the neutral 2-[(pyrazin-2-yl­amino)­meth­yl]phenol mol­ecule are disordered about the rotation axis in a 1:1 ratio. These two are connected by a pyrazine–pyrazine N^1^—H⋯N^4^ hydrogen bond. The cation, whose two aromatic rings are twisted along the –CH_2_—NH– bond by 76.8 (1)°, is a hydrogen-bond donor to the perchlorate ion through the N atom of this link.

## Related literature

For 2-{[(pyrazin-2-yl)amino]­meth­yl}phenol, see: Gao & Ng (2012 ▶).
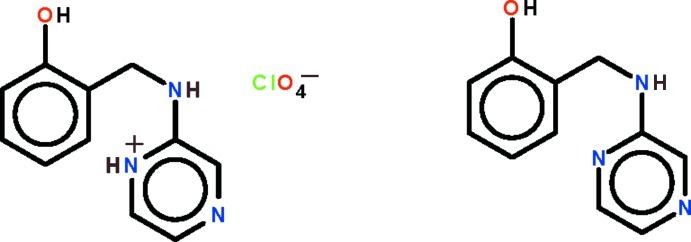


## Experimental

### 

#### Crystal data

C_11_H_12_N_3_O^+^·ClO_4_^−^·C_11_H_11_N_3_O
*M* *_r_* = 502.91
Monoclinic, 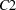

*a* = 19.3402 (14) Å
*b* = 5.9467 (3) Å
*c* = 11.1761 (9) Å
β = 116.263 (10)°
*V* = 1152.68 (16) Å^3^
*Z* = 2
Mo *K*α radiation
μ = 0.22 mm^−1^
*T* = 295 K
0.24 × 0.21 × 0.18 mm

#### Data collection

Agilent Technologies Excalibur Eos diffractometer
Absorption correction: multi-scan (*CrysAlis PRO*; Agilent, 2012 ▶) *T*_min_ = 0.950, *T*_max_ = 0.962
4085 measured reflections
2272 independent reflections
1859 reflections with *I* > 2σ(*I*)
*R*_int_ = 0.017

#### Refinement

*R*[*F* ^2^ > 2σ(*F* ^2^)] = 0.047
*wR*(*F* ^2^) = 0.116
*S* = 1.02
2272 reflections
188 parameters
49 restraints
H atoms treated by a mixture of independent and constrained refinement
Δρ_max_ = 0.25 e Å^−3^
Δρ_min_ = −0.29 e Å^−3^
Absolute structure: Flack (1983 ▶), 970 Friedel pairs
Flack parameter: 0.08 (12)

### 

Data collection: *CrysAlis PRO* (Agilent, 2012 ▶); cell refinement: *CrysAlis PRO*; data reduction: *CrysAlis PRO*; program(s) used to solve structure: *SHELXS97* (Sheldrick, 2008 ▶); program(s) used to refine structure: *SHELXL97* (Sheldrick, 2008 ▶); molecular graphics: *X-SEED* (Barbour, 2001 ▶); software used to prepare material for publication: *publCIF* (Westrip, 2010 ▶).

## Supplementary Material

Crystal structure: contains datablock(s) global, I. DOI: 10.1107/S1600536812031558/xu5583sup1.cif

Structure factors: contains datablock(s) I. DOI: 10.1107/S1600536812031558/xu5583Isup2.hkl

Supplementary material file. DOI: 10.1107/S1600536812031558/xu5583Isup3.cml

Additional supplementary materials:  crystallographic information; 3D view; checkCIF report

## Figures and Tables

**Table 1:** Hydrogen-bond geometry (Å, °)

*D*—H⋯*A*	*D*—H	H⋯*A*	*D*⋯*A*	*D*—H⋯*A*
O1—H1⋯N1^i^	0.84 (1)	1.99 (2)	2.813 (3)	168 (5)
N2—H2⋯N2^ii^	0.88 (1)	1.93 (2)	2.793 (5)	166 (6)
N3—H3⋯O2	0.88 (1)	2.04 (2)	2.868 (7)	158 (4)

Symmetry codes: (i) 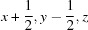; (ii) 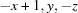.

**Fig. 1.**
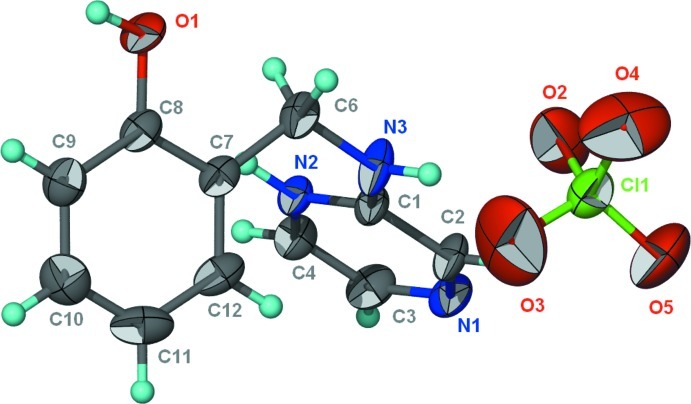
Thermal ellipsoid plot (Barbour, 2001) of C11H12N3O+.ClO4-.C11H11N3O at the 50% probability level; hydrogen atoms are drawn as spheres of arbitrary radius. The disorder is not shown.

## supplementary crystallographic information

### Comment

Salicylaldehyde condenses with romatic amines to yield Schiff bases, which serve as chelating ligands to a plethora of metal systems. These Schiff bases can be readily reduce to the corresponding secondary amines, which can also function as chelating ligands. Curiously, there are only few 2-(arylamino)methylphenols compared with the plethora of Schiff bases in the chemical literature. We have communicated the crystal structure of 2-{[(pyrazin-2-yl)amino]methyl}phenol (Gao & Ng, 2012). The compound reacts with half a molar equivalent of perchloric acid to form the co-crystal, C_11_H_12_N_3_O^+^.ClO_4_^-^.C_11_H_11_N_3_ (Scheme I). The C_11_H_12_N_3_O^+^ cation and the neutral C_11_H_11_N_3_O molecule are disordered about the same position in a 1:1 ratio. The crystal structure is interpreted in terms of the cation and neutral molecule being an N_1-pyrazine_–*H*···N_4-pyrazine_ hydrogen bond. The cation, whose two aromatic rings are twisted along the –CH_2_–NH– bond by 76.8 (1) °, is hydrogen-bond donor to the perchlorate ion through the N atom of this link.

### Experimental

A solution of 2-aminopyrazine (1 mmol) and salicylaldehyde (1 mmol) in toluene (50 ml) was heated for 10 h. The solvent was removed under vacuum, and the residue was reduced in absolute methanol by sodium borohydride. Light yellow crystals were obtained by recrystallization from methanol to which several drops of perchloric acid were added.

### Refinement

Carbon-bound H-atoms were placed in calculated positions [C–H 0.93 to 0.97 Å, *U*_iso_(H) 1.2*U*_eq_(C)] and were included in the refinement in the riding model approximation.

The amino/pyrazinium and hydroxy H-atoms were located in a difference Fourier map, and were refined with distance restraints of N–H 0.88±0.01, O–H 0.84±0.01 Å; the temperature factors of the hydroxy amino H atoms were refined. The pyrazinium H atom should have only half-site occupancy; its temperature factor could not be refined and it was instead tied by a factor of 1.2 times.

The perchlorate ion is disordered about a twofold rotation axis; the chlorine atom itself is ordered. The four Cl–O distances were restrained to within 0.01 Å of each other as were the O···O distances. The temperature factors of the O atoms were tightly restrained to be nearly isotropic.

### Crystal data

**Table:**

C_11_H_12_N_3_O^+^·ClO_4_^−^·C_11_H_11_N_3_O	*F*(000) = 524
*M**_r_* = 502.91	*D*_x_ = 1.449 Mg m^−^^3^
Monoclinic, *C*2	Mo *K*α radiation, λ = 0.71073 Å
Hall symbol: C 2y	Cell parameters from 1220 reflections
*a* = 19.3402 (14) Å	θ = 3.6–26.3°
*b* = 5.9467 (3) Å	µ = 0.22 mm^−^^1^
*c* = 11.1761 (9) Å	*T* = 295 K
β = 116.263 (10)°	Prism, faint yellow
*V* = 1152.68 (16) Å^3^	0.24 × 0.21 × 0.18 mm
*Z* = 2	

### Data collection

**Table:**

Agilent Technologies Excalibur Eos diffractometer	2272 independent reflections
Radiation source: Enhance (Mo) X-ray Source	1859 reflections with *I* > 2σ(*I*)
Graphite monochromator	*R*_int_ = 0.017
Detector resolution: 16.1954 pixels mm^-1^	θ_max_ = 26.4°, θ_min_ = 3.6°
ω scan	*h* = −24→18
Absorption correction: multi-scan (*CrysAlis PRO*; Agilent, 2012)	*k* = −7→6
*T*_min_ = 0.950, *T*_max_ = 0.962	*l* = −13→13
4085 measured reflections	

### Refinement

**Table:**

Refinement on *F*^2^	Secondary atom site location: difference Fourier map
Least-squares matrix: full	Hydrogen site location: inferred from neighbouring sites
*R*[*F*^2^ > 2σ(*F*^2^)] = 0.047	H atoms treated by a mixture of independent and constrained refinement
*wR*(*F*^2^) = 0.116	*w* = 1/[σ^2^(*F*_o_^2^) + (0.0572*P*)^2^ + 0.4309*P*] where *P* = (*F*_o_^2^ + 2*F*_c_^2^)/3
*S* = 1.02	(Δ/σ)_max_ < 0.001
2272 reflections	Δρ_max_ = 0.25 e Å^−^^3^
188 parameters	Δρ_min_ = −0.29 e Å^−^^3^
49 restraints	Absolute structure: Flack (1983), 970 Friedel pairs
Primary atom site location: structure-invariant direct methods	Flack parameter: 0.08 (12)

### Special details

**Table:**

Geometry. All e.s.d.'s (except the e.s.d. in the dihedral angle between two l.s. planes) are estimated using the full covariance matrix. The cell e.s.d.'s are taken into account individually in the estimation of e.s.d.'s in distances, angles and torsion angles; correlations between e.s.d.'s in cell parameters are only used when they are defined by crystal symmetry. An approximate (isotropic) treatment of cell e.s.d.'s is used for estimating e.s.d.'s involving l.s. planes.

### Fractional atomic coordinates and isotropic or equivalent isotropic displacement parameters (Å^2^)

**Table:**

	*x*	*y*	*z*	*U*_iso_*/*U*_eq_	Occ. (<1)
Cl1	0.5000	0.4267 (2)	0.5000	0.0579 (4)	
O1	0.69568 (12)	0.7165 (4)	0.1561 (3)	0.0532 (6)	
H1	0.7398 (12)	0.706 (9)	0.160 (4)	0.096 (16)*	
O2	0.4621 (4)	0.4483 (13)	0.3574 (4)	0.097 (2)	0.50
O3	0.5616 (4)	0.5734 (14)	0.5459 (9)	0.157 (4)	0.50
O4	0.5237 (5)	0.2039 (9)	0.5269 (9)	0.139 (4)	0.50
O5	0.4483 (5)	0.4823 (15)	0.5488 (10)	0.133 (4)	0.50
N1	0.33389 (14)	1.1417 (5)	0.1364 (3)	0.0477 (7)	
N2	0.45243 (14)	1.0855 (4)	0.0644 (2)	0.0356 (6)	
H2	0.489 (3)	1.095 (11)	0.037 (6)	0.043*	0.50
N3	0.50166 (16)	0.7989 (5)	0.2224 (3)	0.0521 (8)	
H3	0.493 (2)	0.719 (7)	0.280 (3)	0.077 (13)*	
C1	0.44928 (15)	0.9564 (5)	0.1601 (3)	0.0364 (7)	
C2	0.38666 (17)	0.9909 (6)	0.1934 (3)	0.0463 (8)	
H2A	0.3837	0.9008	0.2590	0.056*	
C3	0.34042 (18)	1.2745 (6)	0.0433 (3)	0.0494 (9)	
H3A	0.3046	1.3885	0.0035	0.059*	
C4	0.39772 (16)	1.2451 (6)	0.0069 (3)	0.0432 (8)	
H4	0.3996	1.3364	−0.0592	0.052*	
C6	0.56937 (16)	0.7511 (6)	0.2008 (3)	0.0437 (7)	
H6A	0.5534	0.7423	0.1055	0.052*	
H6B	0.5896	0.6049	0.2387	0.052*	
C7	0.63300 (16)	0.9214 (6)	0.2598 (3)	0.0373 (6)	
C8	0.69713 (16)	0.8951 (6)	0.2350 (3)	0.0402 (7)	
C9	0.75839 (19)	1.0441 (6)	0.2873 (3)	0.0487 (8)	
H9	0.8011	1.0232	0.2710	0.058*	
C10	0.7561 (2)	1.2220 (7)	0.3631 (3)	0.0577 (9)	
H10	0.7969	1.3233	0.3973	0.069*	
C11	0.6936 (2)	1.2508 (7)	0.3886 (3)	0.0602 (10)	
H11	0.6923	1.3715	0.4404	0.072*	
C12	0.6323 (2)	1.1010 (6)	0.3376 (3)	0.0497 (9)	
H12	0.5903	1.1219	0.3560	0.060*	

### Atomic displacement parameters (Å^2^)

**Table:**

	*U*^11^	*U*^22^	*U*^33^	*U*^12^	*U*^13^	*U*^23^
Cl1	0.0570 (7)	0.0717 (9)	0.0516 (7)	0.000	0.0300 (6)	0.000
O1	0.0385 (12)	0.0598 (15)	0.0739 (16)	0.0029 (12)	0.0364 (12)	−0.0173 (13)
O2	0.129 (5)	0.104 (5)	0.049 (3)	−0.032 (5)	0.030 (3)	0.005 (4)
O3	0.070 (4)	0.208 (9)	0.164 (8)	−0.059 (5)	0.024 (5)	−0.032 (7)
O4	0.186 (10)	0.093 (5)	0.134 (8)	0.077 (6)	0.068 (7)	0.035 (5)
O5	0.149 (7)	0.155 (7)	0.158 (7)	−0.022 (6)	0.126 (6)	−0.036 (6)
N1	0.0338 (13)	0.0600 (18)	0.0561 (16)	0.0066 (13)	0.0261 (13)	−0.0047 (15)
N2	0.0327 (13)	0.0398 (14)	0.0403 (13)	0.0037 (11)	0.0216 (11)	0.0041 (11)
N3	0.0438 (15)	0.0585 (19)	0.071 (2)	0.0188 (14)	0.0405 (15)	0.0297 (16)
C1	0.0305 (14)	0.0414 (16)	0.0445 (16)	0.0024 (14)	0.0231 (12)	0.0018 (15)
C2	0.0391 (17)	0.058 (2)	0.056 (2)	0.0027 (16)	0.0337 (15)	0.0047 (16)
C3	0.0413 (17)	0.056 (2)	0.0513 (19)	0.0182 (17)	0.0210 (15)	0.0041 (17)
C4	0.0421 (16)	0.0475 (18)	0.0392 (16)	0.0081 (16)	0.0172 (14)	0.0095 (15)
C6	0.0374 (15)	0.0441 (18)	0.0576 (19)	0.0147 (15)	0.0283 (14)	0.0126 (16)
C7	0.0371 (14)	0.0404 (16)	0.0362 (14)	0.0121 (15)	0.0179 (12)	0.0082 (15)
C8	0.0353 (16)	0.0486 (19)	0.0389 (15)	0.0111 (15)	0.0184 (13)	0.0056 (15)
C9	0.0408 (18)	0.057 (2)	0.0479 (19)	0.0026 (15)	0.0195 (16)	0.0043 (16)
C10	0.053 (2)	0.061 (2)	0.047 (2)	−0.0041 (19)	0.0118 (17)	0.0012 (19)
C11	0.072 (2)	0.056 (2)	0.0398 (18)	0.014 (2)	0.0133 (17)	−0.0103 (16)
C12	0.053 (2)	0.058 (2)	0.0429 (18)	0.0198 (18)	0.0262 (16)	0.0046 (16)

### Geometric parameters (Å, º)

**Table:**

Cl1—O5^i^	1.375 (6)	C1—C2	1.431 (4)
Cl1—O5	1.375 (6)	C2—H2A	0.9300
Cl1—O3	1.380 (5)	C3—C4	1.350 (5)
Cl1—O3^i^	1.380 (5)	C3—H3A	0.9300
Cl1—O4^i^	1.390 (5)	C4—H4	0.9300
Cl1—O4	1.390 (5)	C6—C7	1.503 (5)
Cl1—O2^i^	1.435 (4)	C6—H6A	0.9700
Cl1—O2	1.435 (4)	C6—H6B	0.9700
O1—C8	1.373 (4)	C7—C12	1.381 (4)
O1—H1	0.840 (11)	C7—C8	1.394 (4)
N1—C2	1.295 (4)	C8—C9	1.385 (4)
N1—C3	1.356 (4)	C9—C10	1.369 (5)
N2—C1	1.339 (4)	C9—H9	0.9300
N2—C4	1.353 (4)	C10—C11	1.368 (5)
N2—H2	0.883 (11)	C10—H10	0.9300
N3—C1	1.328 (4)	C11—C12	1.388 (5)
N3—C6	1.461 (4)	C11—H11	0.9300
N3—H3	0.877 (11)	C12—H12	0.9300
			
O5—Cl1—O3	111.1 (4)	N2—C4—H4	119.3
O5—Cl1—O4	111.9 (5)	N3—C6—C7	114.6 (3)
O3—Cl1—O4	111.9 (4)	N3—C6—H6A	108.6
O5—Cl1—O2	108.4 (4)	C7—C6—H6A	108.6
O3—Cl1—O2	106.8 (4)	N3—C6—H6B	108.6
O4—Cl1—O2	106.4 (4)	C7—C6—H6B	108.6
C8—O1—H1	107 (4)	H6A—C6—H6B	107.6
C2—N1—C3	117.4 (3)	C12—C7—C8	118.0 (3)
C1—N2—C4	118.6 (2)	C12—C7—C6	124.3 (3)
C1—N2—H2	130 (4)	C8—C7—C6	117.7 (3)
C4—N2—H2	111 (4)	O1—C8—C9	122.4 (3)
C1—N3—C6	125.6 (3)	O1—C8—C7	116.6 (3)
C1—N3—H3	115 (3)	C9—C8—C7	121.0 (3)
C6—N3—H3	120 (3)	C10—C9—C8	120.0 (3)
N3—C1—N2	121.9 (2)	C10—C9—H9	120.0
N3—C1—C2	120.1 (3)	C8—C9—H9	120.0
N2—C1—C2	117.9 (3)	C11—C10—C9	119.9 (4)
N1—C2—C1	123.0 (3)	C11—C10—H10	120.0
N1—C2—H2A	118.5	C9—C10—H10	120.0
C1—C2—H2A	118.5	C10—C11—C12	120.5 (3)
C4—C3—N1	121.6 (3)	C10—C11—H11	119.8
C4—C3—H3A	119.2	C12—C11—H11	119.8
N1—C3—H3A	119.2	C7—C12—C11	120.7 (3)
C3—C4—N2	121.4 (3)	C7—C12—H12	119.7
C3—C4—H4	119.3	C11—C12—H12	119.7
			
C6—N3—C1—N2	2.6 (5)	N3—C6—C7—C8	−175.6 (3)
C6—N3—C1—C2	−178.1 (3)	C12—C7—C8—O1	−179.2 (3)
C4—N2—C1—N3	−179.0 (3)	C6—C7—C8—O1	1.2 (4)
C4—N2—C1—C2	1.7 (4)	C12—C7—C8—C9	0.3 (4)
C3—N1—C2—C1	−1.5 (5)	C6—C7—C8—C9	−179.3 (3)
N3—C1—C2—N1	179.9 (3)	O1—C8—C9—C10	178.6 (3)
N2—C1—C2—N1	−0.7 (5)	C7—C8—C9—C10	−0.9 (5)
C2—N1—C3—C4	2.8 (5)	C8—C9—C10—C11	0.8 (5)
N1—C3—C4—N2	−1.8 (5)	C9—C10—C11—C12	−0.2 (5)
C1—N2—C4—C3	−0.5 (5)	C8—C7—C12—C11	0.3 (5)
C1—N3—C6—C7	74.2 (4)	C6—C7—C12—C11	179.9 (3)
N3—C6—C7—C12	4.9 (4)	C10—C11—C12—C7	−0.4 (5)

Symmetry code: (i) −*x*+1, *y*, −*z*+1.

### Hydrogen-bond geometry (Å, º)

**Table:**

*D*—H···*A*	*D*—H	H···*A*	*D*···*A*	*D*—H···*A*
O1—H1···N1^ii^	0.84 (1)	1.99 (2)	2.813 (3)	168 (5)
N2—H2···N2^iii^	0.88 (1)	1.93 (2)	2.793 (5)	166 (6)
N3—H3···O2	0.88 (1)	2.04 (2)	2.868 (7)	158 (4)

Symmetry codes: (ii) *x*+1/2, *y*−1/2, *z*; (iii) −*x*+1, *y*, −*z*.
